# Calcium-Activated Potassium Channels BK and IK1 Are Functionally Expressed in Human Gliomas but Do Not Regulate Cell Proliferation

**DOI:** 10.1371/journal.pone.0012304

**Published:** 2010-08-20

**Authors:** Iskandar F. Abdullaev, Alena Rudkouskaya, Alexander A. Mongin, Yu-Hung Kuo

**Affiliations:** 1 Center for Neuropharmacology and Neuroscience, Albany Medical College, Albany, New York, United States of America; 2 Division of Neurosurgery, Albany Medical College, Albany, New York, United States of America; Okayama University Graduate School of Medicine, Dentistry and Pharmaceutical Sciences, Japan

## Abstract

Gliomas are morbid brain tumors that are extremely resistant to available chemotherapy and radiology treatments. Some studies have suggested that calcium-activated potassium channels contribute to the high proliferative potential of tumor cells, including gliomas. However, other publications demonstrated no role for these channels or even assigned them antitumorogenic properties. In this work we characterized the expression and functional contribution to proliferation of Ca^2+^-activated K^+^ channels in human glioblastoma cells. Quantitative RT-PCR detected transcripts for the big conductance (BK), intermediate conductance (IK1), and small conductance (SK2) K^+^ channels in two glioblastoma-derived cell lines and a surgical sample of glioblastoma multiforme. Functional expression of BK and IK1 in U251 and U87 glioma cell lines and primary glioma cultures was verified using whole-cell electrophysiological recordings. Inhibitors of BK (paxilline and penitrem A) and IK1 channels (clotrimazole and TRAM-34) reduced U251 and U87 proliferation in an additive fashion, while the selective blocker of SK channels UCL1848 had no effect. However, the antiproliferative properties of BK and IK1 inhibitors were seen at concentrations that were higher than those necessary to inhibit channel activity. To verify specificity of pharmacological agents, we downregulated BK and IK1 channels in U251 cells using gene-specific siRNAs. Although siRNA knockdowns caused strong reductions in the BK and IK1 current densities, neither single nor double gene silencing significantly affected rates of proliferation. Taken together, these results suggest that Ca^2+^-activated K^+^ channels do not play a critical role in proliferation of glioma cells and that the effects of pharmacological inhibitors occur through their off-target actions.

## Introduction

Gliomas are primary brain tumors that arise from glial cells. They represent 30 to 60% of CNS primary tumors, with the incidence of 2 to 3 new cases per 100,000 persons annually [1], [2]. Based on their histopathological characteristics, gliomas are classified as grades I through IV, with higher grades being more de-differentiated and malignant [3], [2]. Grade IV gliomas, also known as glioblastoma multiforme (GBM), have a very high proliferative potential, are invasive and resistant to currently available therapies [3], [4]. Only 30% of GBM patients survive one year, and the average life expectancy remains approximately 14–18 months even with maximal therapy including gross total surgical resection followed by chemotherapy and radiation therapy [5], [6]. Despite medical advances over the past 30 years, there has not been a significant impact on GBM patient survival [4]. Therefore, a better understanding GBM biology is needed in order to develop novel therapeutic treatments for this disease.

There is extensive literature suggesting that proliferation of normal and transformed animal cells requires activity of certain potassium (K^+^) channels (see for example [7], [8], [9], [10]). K^+^ channels are thought to facilitate progression through cell cycle checkpoints, likely via modulation of membrane potential (reviewed in [11], [12]). Particularly, progression through the G_1_/S checkpoint in many cell types is associated with increased K^+^ channel activity and transient hyperpolarization [12]. Conversely, G_2_/M transition may involve depolarization and be associated with increased Cl^−^ currents [12]. On the other hand, a number of studies suggest that concomitant modulation of K^+^ and Cl^−^ channel activity may lead to cell cycle-dependent changes in cell volume (reviewed in [13], [14]).

One group of K^+^ channels that may be relevant to proliferation of both malignant and non-malignant cells are the Ca^2+^-activated K^+^ channels ([14], see also references below). These channels are subdivided into big conductance (large or maxi-) K^+^ channels (BK), intermediate conductance K^+^ channels (IK1), and small conductance K^+^ channels (SK) [15]. Each of these sub-classes can be discriminated based on their biophysical and pharmacological properties. BK channels, also known as SLO1, are broadly expressed in various tissues but have particular functional significance for membrane potential regulation in excitable and exocrine cells [15], [16]. The pore-forming subunit of BK is encoded by the KCNMA1 gene. BKs are voltage-gated channels that are activated at high positive potentials with a large unitary conductance of 100–300 pS. Increases in the intracellular Ca^2+^ concentration to the micromolar levels strongly shift the voltage-dependence of BK to more negative potentials [16]. Paxilline, penitrem A, iberiotoxin, and low concentrations of tetraethyl ammonium are potent and specific inhibitors of BK [16]. IK1 channels, also known as KCa 3.1 or SK4, are the product of the *KCNN4* gene. They are expressed in a variety of tissues and play diverse physiological roles [15], [17]. Unlike BK, IK1 channels are strictly Ca^2+^ dependent as their channel-forming subunit is constitutively associated with calmodulin [18]. Once activated by cytosolic Ca^2+^, they show an intermediate single channel conductance of 33–42 pS [17]. Clotrimazole and its derivative TRAM-34 are potent inhibitors of IK1, discriminating it from other Ca^2+^-activated K^+^ channels [19], [17].

Prior studies have implicated BK channels in the proliferation and migration of glioblastoma cells [20], [21], [22]. BK has also been associated with growth control in cervical, ovarian, prostate, and breast cancer derived cell lines [23], [24], [25]. However, a number of other studies contradict these findings and suggest that BK channels are not required for proliferation or even have antitumorogenic properties, including in glioma cells [26], [27], [28], [29]. In addition to BK, intermediate conductance Ca^2+^-activated K^+^ channels (IK1) have also been proposed to regulate growth rate in numerous types of malignant and non-malignant mammalian cells [30], [31], [32], [33], [34], [35]. More importantly, the inhibitor of the IK1 clotrimazole strongly reduces tumor load and metastases *in vivo* in several cancer types including GBM [36], [37], [38]. It should be noted, however, that some studies question the functional expression of IK1 in GBM cells [22].

Because of the uncertain functional significance of BK and IK channels in glioblastoma proliferation, in the present study we used a combination of pharmacological and molecular biology tools to explore their functional significance. We found that pharmacological inhibitors of both BK and IK1 strongly suppress glioma cell growth in an additive fashion. However, low concentration of the same blockers that were sufficient to inhibit channel activity had no effect on cell proliferation. To address this discrepancy, we downregulated BK and IK1 channels using gene-specific siRNAs. siRNA transfections caused strong reductions in K^+^ current densities but no changes in cell growth. These data argue against a critical role for BK and IK1 in GBM proliferation.

## Materials and Methods

### Materials

Clotrimazole, paxilline, penitrem A, 8,14-Diaza-1,7(1,4)-diquinolinacyclotetradecaphane trifluoroacetate salt (UCL1848), and all other salts and chemicals were from Sigma-Aldrich (St. Louis, MO, USA) unless otherwise noted. 1-[(2-Chlorophenyl)diphenylmethyl]-1H-pyrazole (TRAM-34) was purchased from Tocris Biosciences (Ellisville, MO, USA). Stock solutions of paxilline (20 mM), penitrem A (20 mM), clotrimazole (30 mM), and TRAM-34 (30 mM) were prepared in DMSO and stored at −30°C. UCL1848 was diluted in water at 20 mM.

### Cell cultures

Two established astrocytoma/glioblastoma cell lines, U251 MG and U87 MG, were used in this study. U251 cells were a gift of Dr. M.G. Kaplitt (Weill-Cornell Medical Center, New York, USA); their exact passage is unknown. U87 cells were obtained from the American Type Culture Collection (ATCC, Manassas, VA, USA) and used at passages 78–90. Both U251 and U87 cells were grown in Dulbecco's Modified Eagle's Medium (DMEM) supplemented with 10% fetal bovine serum (FBS), 50 U/ml penicillin, and 50 mg/ml streptomycin at 37°C in a humidified atmosphere containing 95% air and 5% CO_2_. All cell culture reagents are from Invitrogen-Gibco (Carlsbad, CA, USA). Culture media was changed twice a week and cells were passaged using recombinant protease TrypLE when 90–95% confluency was reached.

Primary glioblastoma cells were prepared from a surgical sample of glioblastoma multiforme. Tissue sample was obtained with written consent under a protocol approved by Albany Medical Center Institutional Review Board. Tumor tissue (∼200–300 mg) was washed twice with ice-cold Ca^2+^-, Mg^2+^-free phosphate-buffered saline (PBS, pH 7.4), minced to small pieces and treated with solution of 0.125% Trypsin/0.015% EDTA in PBS containing 250 µg/ml DNAse I. After brief digestion, tissue fragments were triturated using fire-polished glass Pasteur pipette, and the resulting cell suspension was filtered through a Nylon cell strainer (70 µm, BD Falcon, Bedford, MA, USA). Cell were grown in T75 cell culture flasks in DMEM plus 20% FBS supplemented with 100 U/ml penicillin, and 100 mg/ml streptomycin at 37°C in a humidified atmosphere containing 95% air and 5% CO_2_.

### Analysis of gene expression in tumor samples and cultured cells

Expression of various Ca^2+^-activated K^+^ channels in cultured cells and the surgical GBM tissue sample was analyzed using quantitative RT-PCR. Cellular or tissue mRNA was isolated using the RNAqueous-4PCR kit (Applied Biosystems-Ambion, Austin, Texas, USA) according to the manufacturer's instructions. Cultured cells were grown in a 60-mm dish to 60–80% confluency and lyzed in 700 µl of lysis buffer. The GBM tumor sample (∼100–150 mg) was homogenized in 1 ml lysis buffer provided with the RNAqueous-4PCR kit. Concentration of mRNA in resulting samples was quantified using NanoDrop 1000 (ThermoFisher Scientific, Wilmington, DE, USA). mRNA was converted to cDNA using the iScript cDNA Synthesis kit (Bio-Rad Laboratories, Hercules, CA, USA) according to the manufacturer's instructions. One µg of mRNA was used in each 20-µl cDNA synthesis reaction mix.

Gene expression was quantified by a real-time PCR using the CFX96 Real-Time PCR Detection System (Bio-Rad) and iTaq SYBR Green Supermix kit (Bio-Rad) according to the manufacturer's instructions. One µl of cDNA product prepared as described above was used for each qPCR reaction. Gene expression was analyzed with the gene-specific QuantiTect Primer Assays (Qiagen, Hilden, Germany; see Table 1 for the list of primers). Two housekeeping genes, *GAPDH* and *RPL13A*, were used as internal reference.

**Table 1:** Quantitative primers for real time PCR.

Gene	Sequence	Manufacturer	Catalog number
*RPL13A* (housekeeping)	Proprietary	Qiagen	QT00089915
*GAPDH* (housekeeping)	Proprietary	Qiagen	QT01192646
*KCNMA1* (BK1 or SLO1)	Proprietary	Qiagen	QT00024157
*KCNN1* (SK1)	Proprietary	Qiagen	QT00025375
*KCNN2* (SK2)	Proprietary	Qiagen	QT00016611
*KCNN3* (SK3)	Proprietary	Qiagen	QT00070966
*KCNN4* (IK1)	Proprietary	Qiagen	QT00003780

### Western blot analysis

Protein expression of the IK1 channel was assessed by the Western blot analysis using polyclonal antibody raised against synthetic peptide corresponding to a region of human IK1 (LYDLQQNLSSSHRALEKQIDTLAGKLDALTELLSTALGPRQLPEPSQQSK, Sigma-Aldrich; cat.# AV35098). Whole cell lysates were diluted with a reducing Laemmli buffer. Proteins were separated on 10% polyacrylamide gel followed by transfer onto an Immun-Blot PDVF membrane (Bio-Rad). The membrane was blocked for 1 hr with 5% nonfat milk in Tris-Phosphate buffer containing 0.05% Tween 20 (TBS-T). It was further incubated overnight at 4°C with primary antibody (1∶500 dilution). After five washes for 5 min with TBS-T, membranes were further incubated with horseradish peroxidase-conjugated secondary antibodies (GE Healthcare/Amersham Biosciences, Piscataway, NJ; 1∶10,000 dilution), followed by four TBS-T washes. The horseradish peroxidase signal was detected and digitized using a chemiluminescence ECLplus reagent (GE Healthcare/Amersham Biosciences) and a luminescent image analyzer LAS-4000 (FujiFilm Medical Systems, Stamford, CT).

### Electrophysiology

BK and IK1 currents were recorded employing conventional whole-cell patch clamp technique [39]. Ion currents were recorded using an Axopatch-200B amplifier (Molecular Devices, Sunnyvale, CA, USA). Patch pipettes were prepared from borosilicate glass (Warner Instruments, Hamden, CT, USA) using a P-97 micropipette puller (Sutter Instruments, Novato, CA, USA). Pipette resistances were 2.5–4 MOm when filled with a solution containing (in mM): 135 KCl, 2 MgSO_4_, 10 HEPES, 2.5 ATP-Na, 0.1 EGTA (pH = 7.2, adjusted with KOH). Bath solution contained (in mM): 140 NaCl, 5 KCl, 3 CaCl_2_, 1.2 MgSO_4_, 10 HEPES, 10 glucose (pH = 7.4, adjusted with NaOH). In experiments to detect IK1 currents, calcium concentration in pipette solution was increased to 750 nM by adding CaCl_2_. Free Ca^2+^ levels were calculated using CaBuf software (G. Droogmans, KU Leuven, Leuven, Belgium). In all experiments series resistance was <15 MOm.

BK currents were recorded from a holding potential of −80 mV in response to step pulses of −80 mV to +140 mV in 20 mV increments. BK current development and inhibition was monitored by applying repetitive (every 3 sec) depolarization ramps from −120 mV to +140 mV. Because the pipette solution is nominally Ca^2+^-free, other Ca^2+^ activated K^+^ channels, such as IK1 and SK1-3 are silent under these experimental conditions.

IK1 currents were recorded from a holding potential of −80 mV in response to step pulses from −120 mV to +80 mV in 20 mV increments. Development and inhibition of the Ca^2+^-dependent IK1 currents were monitored by applying repetitive (every 3 sec) depolarization ramps from −120 mV to +80 mV. Since these conditions also favor activation of BK currents, 2 µM paxilline was added to bath solution to isolate the IK1 currents.

### Cell proliferation assays

Cell proliferation rates were quantified using two approaches: Coulter counter technique and colorimetric MTT proliferation assay. U251 and U87 cells were plated in 24-well cell culture plates (TPP, Trasadingen, Switzerland, Europe) at the density of 10,000 cells per well and were allowed to grow overnight. The next day, culture media was replaced with serum-containing or serum-free media including the various pharmacological inhibitors or vehicle controls. Initial cell density was quantified at this time in a separate “baseline” plate prepared in the same fashion. We used two types of culture media in pharmacological experiments: serum-containing (DMEM plus 10% FBS), or serum-free medium prepared using OptiMEM with B27 supplement (Invitrogen; 1∶50 dilution). To preclude the potential degradation of inhibitors, we replaced inhibitor-containing media with freshly prepared every 24 hrs. 48 hrs after first application of inhibitors, extracellular media were removed and cells were processed for proliferation assays.

For MTT assays, cells were briefly washed with basal solution containing (in mM): 135 NaCl, 3.8 KCl, 1.2 MgSO_4_, 1.3 CaCl_2_, 1.2 KH_2_PO_4_, 10 HEPES, and 10 glucose (pH = 7.4, adjusted with NaOH). Aliquots of basal solution containing 0.5 mg/ml thiazolyl blue tetrazolium bromide (MTT, Sigma-Aldrich) were then added to each well. After 30-min incubation at 37°C MTT solution was removed, and cells were solubilized in acidified isopropanol to dissolve newly formed MTT-formazan particles. 250-µl aliquots from each well were transferred into a 96-well plate. Absorbances at 562 nm were measured using ELx800 Absorbance microplate reader (BioTek Instruments, Winooski, VT, USA).

For Coulter counter assays, cells were detached from substrate using recombinant protease TrypLE and counted using a Z1 Series Coulter Counter (Beckman Coulter, Miami, FL, USA).

### siRNA transfections

To downregulate BK and IK1, U251 cells were transfected with gene-specific siRNAs using a Nucleofector II (Lonza, Cologne, Germany) and the Amaxa Nucleofection kit T according to the manufacturer's protocol. Cells were removed from the substrate with TrypLE, counted, centrifuged at 300 g for 10 min, and mixed with nucleofection suspension buffer containing 0.5 µg of GFP cDNA (as a control of transfection efficacy) and 1 µM siRNA of choice. Cells were electroporated using program T-20 and then plated on either 24-well plates for proliferation assays or glass coverslips for patch clamp experiments. We used two to three siRNA constructs per gene that were obtained from Qiagen or Ambion. Target sequences for each siRNA are listed in Table 2. As a negative control we used nonsense AllStars negative control siRNA (Qiagen) or siRNA to an unrelated K^+^ channel TREK.

**Table 2:** Gene-specific siRNA constructs used in the gene downregulation experiments.

Target gene	siRNA #	Sense strand	Antisense strand	Manuf.
Hs_KCNMA1	BK#1	5′-CCUGAAAUCAUAGAGUUAAtt-3′	5′-UUAACUCUAUGAUUUCAGGga-3′	Ambion
Hs_KCNMA1	BK#2	5′-CAUCAAUCUAUGCAGUUUtt-3′	5′-AAACUGCAUAGAUUUGAUGtt-3′	Ambion
Hs_KCNN4	IK1#5	5′-CUUUGUAAUAAAUGUUAAATT-3′	5-UUUAACAUUUAUUACAAAGAT-3′	Qiagen
Hs_KCNN4	IK1#6	5′-CAUCGGCGCUCUCAAUCAATT-3′	5′-UUGAUUGAGAGCGCCGAUGCT-3′	Qiagen
Hs_KCNN4	IK1#7	5′-CCGGAAGCUCCGGGAACAATT-3′	5′-UUGUUCCCGGAGCUUCCGGTG-3′	Qiagen
Hs_KCNK2	TREK1#1	5′-CGCAUCAUCUCAACAAUCAtt-3′	5′-UGAUUGUUGAGAUGAUGCGaa-3′	Ambion
N/A	NC	Proprietary nonsense siRNA (AllStars Negative Control)		Qiagen

N/A, not applicable; NC, negative control; Manuf., manufacturer.

### Statistical analysis

All data presented as mean values ±SE. Number of independent experiments per group is indicated in each figure legend. Statistical significance was determined by one-way analysis of variance (ANOVA) and *a priori* Newman-Keuls test for multiple comparisons. p<0.05 was accepted as significant. In those experiments where experimental values were normalized to controls, statistical difference from controls was calculated with unpaired Student's *t*-test. Origin 6.0 (Origin Labs, Northampton, MA, USA) and Prism 5 (GraphPad Software, San Diego, CA, USA) were used for statistical analysis.

## Results

### Expression of mRNAs encoding the Ca^2+^-activated K^+^ channels BK, IK1 and SK1-3 in glioma cell lines and in a surgical sample of glioblastoma multiforme

**Figure 1:**
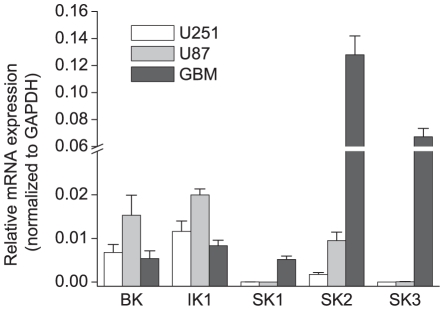
mRNA expression of Ca^2+^-activated K^+^ channels in human gliomas cell lines and in tissue sample of glioblastoma multiforme. Relative levels of mRNA expression for BK, IK1 and SK1-3 channels were quantified in U251, U87, and a GBM tissue sample using reverse transcription quantitative PCR. Expression levels were normalized to the expression of two housekeeping genes, GAPDH (shown in graph) and ribosomal protein RPL13A. Mean values ±SE of gene expression in three independent cell preparations for U251 and U81, and three independent measurements for GBM are shown.

We initially evaluated the expression of Ca^2+^-activated K^+^ channels in glioma cell lines at the mRNA level. U251 and U87 are both derived from human gliomas, but demonstrate different morphological and growth characteristics as well as substantial difference in gene expression profiles [40], [41]. For example, U87 cell express wild type p53, while U251 carry its mutated form [40]. We found expression of BK, IK1, and SK2 transcripts in U251 and U87 cells. The levels of transcripts for each of these channels were quantitatively similar, representing ∼0.5–2% of the mRNA expression levels for the housekeeping gene GAPDH (Fig. 1). SK1 and SK3 expression levels were negligible. In order to determine if glioma cells *in situ* have a similar Ca^2+^-activated K^+^ channel expression profile, we isolated mRNA from a surgical sample that was histopathologically identified as a GBM. In the GBM sample, BK and IK1 mRNA expression levels were comparable to those in U251 and U87 cells (Fig. 1). However, GBM SK2 transcript levels were substantially higher than in glioma cell lines. Additionally, we found the expression of SK1 and SK3 in the surgical sample that was not present in the glioma cell lines (Fig. 1).

### Functional expression of BK and IK1 in glioma cell lines and in primary GBM cells

Expression of mRNA does not necessarily result in production of functional plasmalemmal channels. For example, Weaver *et al.* found high levels of the IK1 mRNA but no IK1 immunoreactive signal in Western blot analysis or functional IK1 currents in electrophysiological experiments in D54 and U251 glioma cell lines [22]. Therefore, we assessed the functional expression of Ca^2+^-activated K^+^ channels using conventional patch clamp technique.

**Figure 2:**
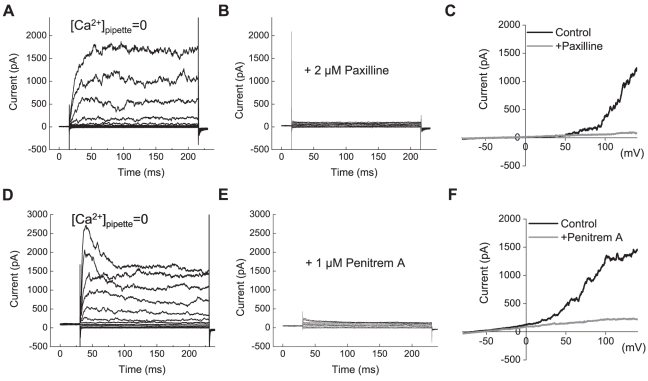
BK channels are functionally expressed in U251 glioma cells. (**A**) Representative recordings of the whole-cell BK currents elicited by depolarization step pulses from −80 mV to +140 mV in 20 mV intervals. To prevent concomitant activation of IK1 and SK channels, no Ca^2+^ was added into pipette solution. (**B**) In the same cell shown in (**A**), whole-cell K^+^ currents were potently suppressed by the specific inhibitor of BK channels paxilline (2 µM). (**C**) Whole cell BK currents in response to depolarization ramps from −80 mV to +140 mV. Representative traces in the absence or presence of paxilline are shown. (**D–F**) Results of similar experiments employing another selective BK blocker penitrem A (1 µM).

When U251 cells were dialyzed with a pipette solution containing no Ca^2+^, we registered large macroscopic K^+^ currents that were activated in response to depolarizing step pulses >40 mV (Fig. 2A, D). This electrophysiological profile is characteristic of BK channels [16]. Under these Ca^2+^-free recording conditions, the addition of specific BK channel blockers paxilline (2 µM, Fig. 2B and C) or penitrem A (1 µM, Fig. 2E and F) nearly completely suppressed transmembrane K^+^ currents. Paxilline and penitrem A data were corroborated with the BK peptide inhibitor charybdotoxin (Fig. S1). Average BK current density, calculated as the paxilline-sensitive component of macroscopic K^+^ currents at +140 mV, was 67±9 pA/pF (n = 11). U87 cells also exhibited BK currents of similar amplitude (data not shown).

**Figure 3:**
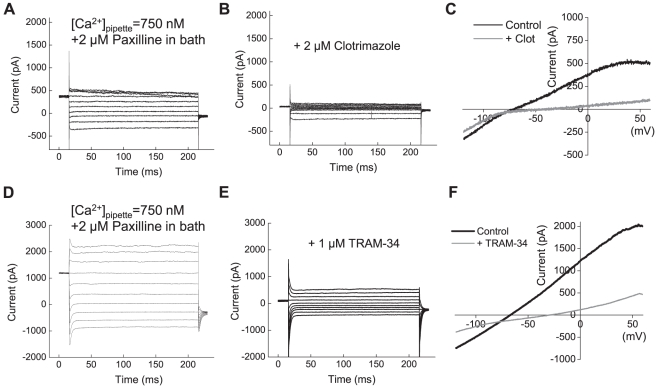
IK1 channels are functionally expressed in U251 glioma cells. (**A**) Representative recordings of the whole-cell IK1 currents elicited by depolarization step pulses from −120 mV to +80 mV in 20 mV intervals. Whole cell IK1 currents were activated by clamping pipette [Ca^2+^] at 750 nM. To prevent concomitant activation of BK currents, 2 µM paxilline was added into bath solution. (**B**) In the same cell shown in (**A**), the whole cell IK1 currents were inhibited by the potent IK1 blocker clotrimazole (2 µM). (**C**) Whole cell currents in response to depolarization ramps from −120 mV to +80 mV in the absence or presence of clotrimazole. (**D–F**) Results of similar experiments employing the selective IK1 blocker TRAM-34 (1 µM).

To record IK1 currents, we increased [Ca^2+^] in the pipette to 750 nM and applied step pulses from –120 mV to +80 mV in 20 mV increments. To prevent concomitant activation of the BK currents, 2 µM paxilline was included in the bath solution. Under these recording conditions, we observed macroscopic K^+^ currents that were saturated at voltages ≥40 mV, consistent with the electrophysiological profile of IK1 channels (Fig. 2A and C) [35]. The specific blockers of IK1 channels 2 µM clotrimazole (Fig. 2B and C), and 1 µM TRAM-34 (Fig. 3E and F) inhibited the K^+^ currents, indicating that they are indeed mediated by the IK1 channels. In U251 cells, average IK1 current density, calculated as the clotrimazole-sensitive component, was 25±6 pA/pF at 0 mV (n = 6). Similar IK1 currents were recorded in U87 cells (Fig. S2).

**Figure 4:**
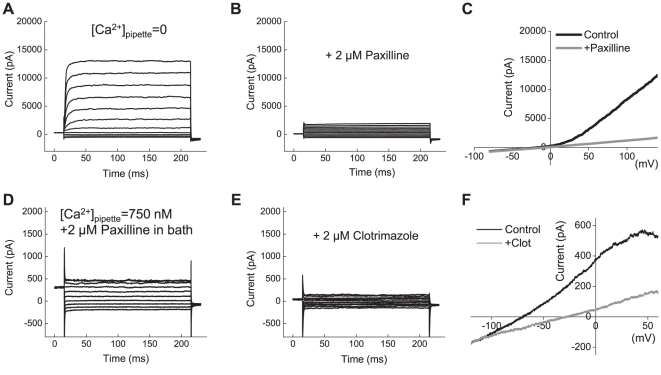
Functional expression of BK and IK1 channels in primary cells derived from glioblastoma multiforme (GBM). (**A**) Representative recordings of macroscopic BK currents in primary cells cultured from surgical sample of glioblastoma multiforme. Currents were elicited by step pulses from −80 mV to +140 mV. (**B**) In the same cell shown in (**A**), the BK blocker paxilline (2 µM) potently inhibited macroscopic K^+^ currents. (**C**) Representative traces of K^+^ currents elicited in response to depolarization ramps from −80 mV to +140 mV in the absence and presence of paxilline. (**D**) Representative recordings of the whole-cell IK1 currents activated by step pulses from −120 to +60 mV. To isolate IK1 currents, [Ca^2+^]_pipette_ was clamped at 750 nM and 2 µM paxilline was added into bath solution. (**E**) The specific IK1 inhibitor clotrimazole (2 µM) potently suppressed macroscopic K^+^ currents. (**F**) Representative traces of K^+^ currents elicited in response to depolarization ramps −120 mV to +60 mV in the absence and presence of clotrimazole. For additional experimental details, see legend to Fig. 2 and Results section.

Since immortalized cell lines frequently have different gene expression profile from cells *in situ* and primary cell cultures, we performed recordings in primary glioma cultures prepared from a human surgical sample of GBM. As shown in Fig. 4, primary GBM cells also exhibited large paxilline-sensitive BK currents under Ca^2+^-free conditions (Fig. 4A–C). Five out of five recorded cells had BK currents, with average current density 80±33 pA/pF at +140 mV. We also observed the clotrimazole-sensitive IK1 currents when [Ca^2+^]_i_ was clamped at 750 nM and 2 µM paxilline was added to the bath solution (Fig. 4D–F). Three out of six recorded GBM cells showed IK1 currents with an average current density of 13±6 pA/pF at 0 mV. The reasons for heterogeneity of IK1 current expression are not clear. However, when observed, GBM IK1 current densities were not statistically different from those found in U251 cells.

Our finding of the functional IK1 currents in all glial cell lines was in conflict with the previous observations of Weaver et al. [22]. Therefore, we performed additional Western Blot analysis of the IK1 protein expression in U251, U87 and GBM cells. Using commercially available polyclonal IK1 antibody, we identified a weak immunopositive band of ∼46–48 kDa in protein lysates prepared from all three cell types (Fig. S3). This molecular weight is very close to the predicted molecular weight of the IK1 protein (45 kDa).

### Effects of BK and IK1 channel blockers on proliferation of U251 and U87 cells

**Figure 5:**
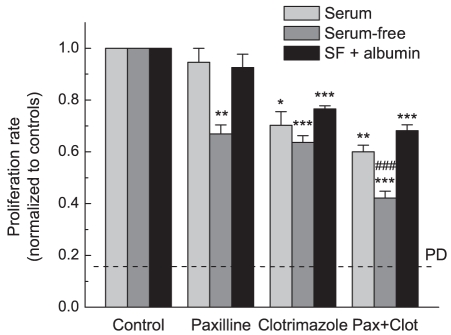
Effects of the BK blocker paxilline and the IK blocker clotrimazole on U251 cell proliferation in serum-containing and serum-free media. Paxilline (10 µM) and clotrimazole (10 µM) were added to culture media alone or in combination, and rates of cell proliferation were determined 48 hrs later using an MTT proliferation assay. Proliferation assays were performed in standard cell culture medium (DMEM +10% FBS), serum-free OptiMEM medium supplemented with serum substitute B27, or in OptiMEM+B27 additionally containing bovine serum albumin (2 mg/ml). Quantitatively similar data were obtained using Coulter counter assay (see Fig. S2). *, p<0.05, **p<0.01, ***p<0.001 vs. control; ^##^p<0.01, combination of drugs vs. paxilline or clotrimazole alone.

**Figure 6:**
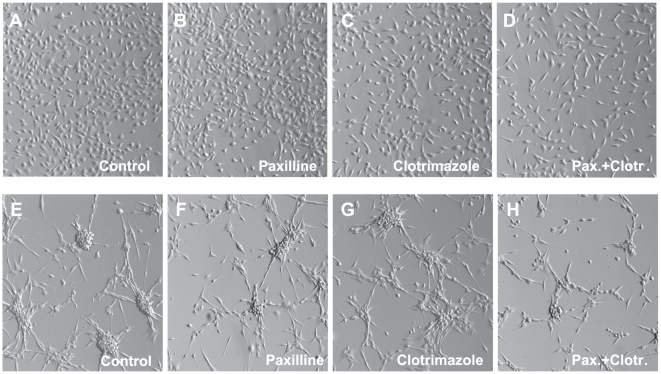
Representative micrographs of U251 (A-D) and U87 (E-H) cells grown in the presence of BK and IK1 blockers. Cells were grown in the serum-free OptiMEM media supplemented with serum substitute B27. The BK blocker paxilline (10 µM) and the IK1 inhibitor clotrimazole (10 µM) were added as indicated. Images of the cells were captured ∼48 hrs after addition of channel blockers using Hoffman modulation contrast optics in Olympus IX71 microscope at 10×10 magnification.

To explore functional involvement of BK and IK1 channels in proliferation, we tested the effects of pharmacological inhibitors of BK and IK1 using two different techniques, Coulter counter and MTT proliferation assays. Using two alternative approaches was important because MTT conversion into light-absorbing formazan is mediated by mitochondrial dehydrogenases and may be affected without changes in cell numbers or viability [42]. In the FBS-containing media, the BK inhibitor paxilline (10 µM) had no effect on cell growth, while the IK1 blocker clotrimazole (10 µM) moderately suppressed cell proliferation of U251 (Fig. 5). The combination of these two drugs caused a significant reduction of cell proliferation as measured by both MTT and Coulter counter assays (Fig. 5 and Fig. S4A). To test whether serum components may reduce the inhibitory effects of channel blockers, we performed additional proliferation assays under serum-free conditions. In OptiMEM medium supplemented with serum substitute B27, U251 proliferation rates were similar to those in cells cultured in DMEM plus 10% FBS. In both media we observed approximately six-fold increases in cell numbers over 48 hrs (data of Coulter counter assays). Under serum-free conditions the efficacy of paxilline was dramatically increased (Fig. 5 and Fig. S4B). Furthermore, the combination of paxilline and clotrimazole produced strong and additive inhibition of cell growth, reducing it by approximately 70% (Fig. 5, Fig. S4B). The combination of drugs also induced strong changes in cell morphology (see Fig. 6A–D). Qualitatively similar data were obtained in U87 cells, although the latter cells were less sensitive to the actions of clotrimazole (see Fig. S5 and micrographs in Fig. 6E–H). To test if drug bio-availability was reduced by the major serum component albumin, we supplemented serum-free media (OptiMEM+B27) with 2 mg/ml bovine serum albumin. Addition of albumin reverted the inhibitory properties of paxilline and clotrimazole to the levels seen in serum-containing media, rendering paxilline completely ineffective (Fig. 5).

**Figure 7:**
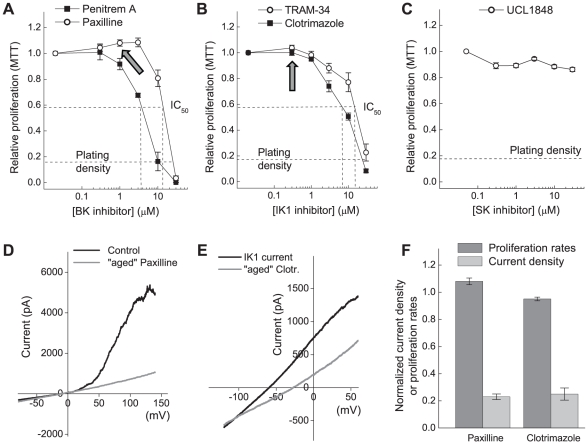
Blockers of the Ca^2+^-activated potassium channels BK and IK1 suppress proliferation of U251 cells in a dose dependent fashion. (**A**) Dose response curves for the effects of the BK channel inhibitors, paxilline and penitrem A, on proliferation of U251 cells. Arrow indicates concentration of paxilline that was used to block K^+^ currents in the electrophysiology experiments presented in (**D**). (**B**) Dose response curves for the effects of the IK1 channel inhibitors, clotrimazole and TRAM-34, on proliferation of U251 cells. Arrow indicates concentration of clotrimazole that was used to block K^+^ currents in the electrophysiological experiments presented in (**E**). (**C**) Dose response curves for the effects of the selective SK1-3 channel blocker UCL1848 on proliferation of U251 cells. (**D**) Effect of the “aged” paxilline on the whole-cell BK currents. Cell proliferation medium containing 10 µM paxilline was collected after 24-hr incubation in cell proliferation assays, diluted 10-fold with electrophysiological bath solution, and used for inhibiting BK currents. Nominal concentration of paxilline was 1 µM (indicated by arrow in (**A**), to compare proliferation and electrophysiology data). Representative of three experiments. (**E**) Effect of the “aged” clotrimazole on the whole-cell IK1 currents. Cell proliferation medium containing 10 µM clotrimazole was collected after 24-hr incubation in cell proliferation assays, diluted 10-fold, and used for inhibiting IK1 currents. Nominal concentration of clotrimazole was 1 µM (indicated by arrow in (**B**)). Representative of three experiments. (**F**) Comparison of normalized effects of the “aged” paxilline and clotrimazole on the whole cell K^+^ currents to their effects on cell proliferation. Inhibition of BK and IK1 currents was calculated as average inhibition of the whole K^+^ cell currents at +140 and 0 mV for BK and IK1, respectively.

We next tested the dose dependency of the effects of paxilline and clotrimazole on U251 cell proliferation and compared their efficacy to the specific BK inhibitor penitrem A [43] and the selective IK1 blocker TRAM-34 [19]. Since high levels of SK2 message were found in both immortalized cell lines and primary cells, we also tested UCL1848, an inhibitor of SK1, SK2, and SK3 [44]. The BK blockers, paxilline and penitrem A, inhibited U251 proliferation in a dose dependent fashion (Fig. 7). At the highest tested concentration (30 µM), both agents were cytotoxic, i.e. reduced cell numbers below seeding densities (Fig. 7A). The IC_50_ values for both inhibitors were substantially higher than those reported for inhibition of BK currents. In our hands, paxilline blocked cell proliferation by 50% at ∼13 µM (Fig. 7A), whereas the reported IC_50_ for inhibition of the whole-cell currents is ∼30 nM [45]. Penitrem A was somewhat more effective and inhibited proliferation with the IC_50_ of ∼3.5 µM (Fig. 7A), but this value greatly exceeded ∼10 nM concentration required for half-maximal inhibition of the BK activity [45]. The IK1 inhibitors clotrimazole and TRAM-34 reduced cell proliferation by 50% at the concentrations of ∼6 and 14 µM, respectively (Fig. 7B). These values were drastically higher than the IC_50_ of 70 nM (clotrimazole) and 20 nM (TRAM-34) that were obtained in electrophysiological experiments [19]. The specific blocker of the SK1-3 channels UCL1848 showed no effect on U251 cell proliferation (Fig. 7C).

The disparity between the IC_50_ values for inhibition of proliferation and channel activity may be explained by decomposition of tested compounds in culture media, or by nonspecific absorption of the drugs during long incubation. To address this possibility, we tested the inhibitory properties of culture media containing nominally 10 µM of either paxilline or clotrimazole after 24-hr incubation in cell proliferation assays. We diluted aliquots of the inhibitor-containing culture media 10-fold with electrophysiological bath solution and performed whole-cell patch recordings. Such dilution was necessary to minimize differences between cell culture media and bath solution. The final nominal concentrations of paxilline and clotrimazole were 1 µM. As seen in Fig. 7D, E, “aged” paxilline and clotrimazole solutions inhibited BK and IK1 currents by >90% and >70%, respectively. Notably, at the nominal concentration of 1 µM, neither paxilline nor clotrimazole affected cell proliferation (compare to the values indicated by the block arrows in Fig. 7A and B). Thus, the effects of paxilline and clotrimazole (as well as the other inhibitors) on cell proliferation are unlikely related to their actions on BK and IK1 activities.

### Effects of gene-specific siRNAs on BK and IK1 channel activity and cell proliferation

**Figure 8:**
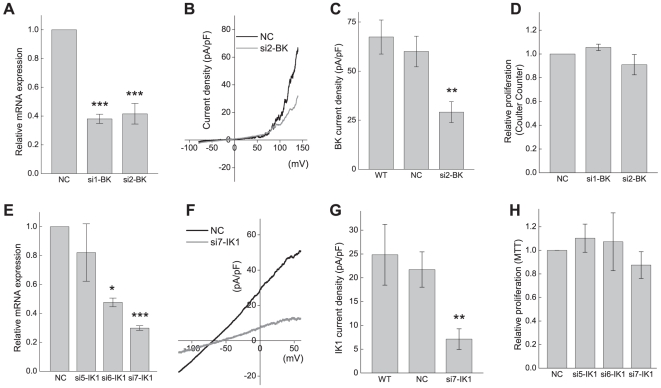
Effects BK and IK1 gene silencing on macroscopic K^+^ current densities and proliferation rates in U251 glioma cells. (**A**) Effect of the BK-specific siRNAs on BK mRNA expression levels. U251 cells were transfected with negative control siRNA (NC) or two different siRNAs targeting BK (si1-BK, and si2-BK). 48 hrs post transfection, mRNA levels were quantified and normalized to housekeeping genes GAPDH and RPL13A using reverse transcription qPCR. Data are the mean values ±SE of 3-5 independent transfections. (**B**) Representative traces of whole-cell BK currents measured at 72 hrs after transfection with negative control siRNA (NC) or siRNA targeting BK (si2-BK). (**C**) Average BK current densities in U251 cells transfected with negative control (NC) or si2-BK siRNAs, as compared to non-transfected cells (WT). BK current density was measured as paxilline-sensitive K^+^ currents at +140 mV. Data are the mean values ±SE from 11-17 cells per group. **p<0.01, vs. negative control and non-transfected cells. (**D**) Effect of transfection with negative control (NC) or BK siRNAs (si1-BK, si2-BK) on proliferation of U251 measured 72 hrs post transfection using Coulter counter assay. Data are the mean values ±SE of 3 independent cell transfections. (**E**) Effect of the IK1-specific siRNAs on IK1 mRNA expression levels. mRNA levels were quantified and normalized to housekeeping genes GAPDH and RPL13A 48 hrs after transfection with negative control siRNA (NC), and three different siRNAs targeting IK1 (si5-IK1, and si6-IK1, si7-IK1. Data are the mean values ±SE in 3 independent transfections. (**F**) Representative whole-cell K^+^ currents measured 72 hrs after transfection with negative control siRNA (NC) or IK1siRNA (si7-IK1). (**G**) Average IK1 current densities in cells transfected with negative control (NC) or si7-IK1 siRNAs, as compared to non-transfected cells (WT). IK1 current density was measured as clotrimazole-sensitive K^+^ currents at 0 mV with 2 µM paxilline added to bath solution. Data are the mean values ±SE from 14-15 cells per experimental group, and n = 6 in WT. **p<0.01, vs. negative control and non-transfected cells. (**H**) Effect of nucleofection with negative control siRNA (NC), or IK1-siRNA (si5-IK1, si6-IK1, si7-IK1) on cell proliferation measured 72 hrs post nucleofection using MTT assay. Data are the mean values ±SE of 3 independent cell transfections.

We decided to further test the functional involvement of BK and IK1 in glioma cell proliferation using an alternative approach. For this purpose, we downregulated BK and IK1 expression using an siRNA technique. At 48 hrs after transfection, the two BK-specific siRNAs significantly downregulated BK mRNA expression levels by 62% and 58% (Fig. 8A, p<0.01). These values likely underestimate the degree of mRNA downregulation in individual cells because only 65–80% of the cells were successfully transfected, as routinely verified by co-transfection with GFP (see Fig. S6). U251 cells transfected with siRNA targeting the unrelated K^+^ channel TREK-1 (*KCNK2*) did not affect levels of BK mRNA expression (n = 3, data not shown). The BK siRNA#2 was used to test for functional suppression of BK channels. As seen in Fig. 8B, C, the BK siRNA#2 induced a 52% decrease in the BK current density 72 hrs after transfection (29±5 pA/pF for BK siRNA vs. 60±8 pA/pF for negative control siRNA, p = 0.002). While the BK siRNA#2 successfully downregulated the BK current densities, neither siRNA#2 nor siRNA#1 affected U251 cell proliferation (Fig. 8D, cell counts performed 72 hrs after transfection).

Two out of three IK1-specific siRNAs tested by us downregulated IK1 mRNA levels by 52% and 70% 48 hrs after transfection, while the third construct proved ineffective (Fig. 8E). The most effective IK1 siRNA construct (siIK1#7) was further tested in electrophysiological functional assays. This siRNA reduced the IK1 current density by 67% 72 hours after transfection (7±2 pA/pF for IK siRNA vs. 21±4 pA/pF in negative control transfections, p = 0.003). As in the case of BK downregulation, the IK1 knockdowns had no effect on proliferation of U251 cells (Fig. 8H).

Because paxilline and clotrimazole reduced cell proliferation in an additive fashion, we explored the effects of simultaneous BK and IK1 downregualtion on rate of U251 cell growth. As in the case of individual channel knockdowns, a combination of the BK siRNA#2 and the IK1 siRNA#7 (two constructs that effectively suppressed BK and IK1 current densities) was ineffective in inhibiting cell growth (Fig. S7).

## Discussion

The main finding of this study is the lack of functional involvement of the Ca^2+^-activated K^+^ channels in proliferation of U251 and U87 glioma cell lines and primary GBM cells. This conclusion is at odds with some previous reports that also used pharmacological agents to assay for functional contribution of BK and/or IK1 to proliferation. Although all the inhibitors of BK and IK1 that we tested in this study suppressed glioma cell proliferation, their effects occurred at concentrations significantly exceeding those needed to block channel function. Downregulation of BK and IK1 activity using siRNA did not cause changes in cell growth. Therefore, the antiproliferative properties of the BK and IK1 channel blockers are likely mediated by off target actions.

BK channels are highly expressed in glioma cell lines and in human glioma tissue [46], [47], [48], [22]. The BK channel expression correlates with glioma malignancy, with higher levels of the BK protein seen in more malignant glioma biopsy samples [48]. BK channels have been implicated in regulation of cell proliferation in a number of malignant cell lines, such as HeLa and A2780 carcinomas, MCF-7 breast cancer, PC-3 prostate cancer, as well as in 1321N1 and D54-MG human glioma lines [20], [21], [23], [24], [25]. These proliferation studies relied on pharmacological inhibitors of BK, such as TEA and iberiotoxin. However, more recent publications, that employed molecular biology techniques and/or BK channel openers, put forward the opposite idea that BK channels may have antiproliferative and antitumorogenic properties. For example, Cambien *et al.* [29] found that silencing of BK channels in human osteosarcoma cells strongly increases tumor load *in vivo*. In A2780 ovarian cancer cells, the BK opener NS1619 produced potent inhibition of cell proliferation [28]. Our own results strongly suggest that BK channels do not play a role in growth of glioma cells *in vitro*. While the BK blockers paxilline and penitrem A inhibited proliferation of U251 cells in a dose-dependent manner, their inhibitory effects were seen at concentrations greatly exceeding those necessary for complete channel block. This points to possible off-target effects of pharmacological agents. Such conclusion was supported by the results of siRNA experiments, in which downregulation of BK channel expression had no impact on U251 proliferation rates. The discrepancy between our BK data and several other reports in glioma cell lines may be explained by different conditions employed in cell proliferation assays. Positive results implicating BK in control of glioma cell growth have been collected using specific cell growth conditions, such as elevated extracellular [K^+^] [20] or serum deprivation [21]. In other malignant cell types, the effects of BK blockers could be seen only when cell growth was stimulated with certain factors such as estradiol [25], [24]. Hence, even if BK may contribute to cell proliferation under specific conditions, its activity is not a prerequisite for cell cycle progression in glioma cells.

A somewhat surprising finding of our work was the detection of functional IK1 channels in two established glioma lines, U251and U87, as well as in primary GBM cells. Although IK1 mRNA is consistently detected in immortalized glioma cell lines and human GBM samples, Weaver *et al.* found no evidence for the IK1 protein expression in numerous glioma samples and were unable to detect whole-cell IK1 currents in U251 cells [22]. Nevertheless, Fioretti *et al.* recently reported functional expression of the IK1 channels in GL-15 and U251 lines and in primary GBM cells [49], [50], [51], similar to findings in the present work. The disparity between these findings and the results of Weaver *et al.* may be due to differences in conditions employed in electrophysiological experiments. In order to isolate the IK1 currents, we and Fioretti *et al.* suppressed the concomitant BK currents by adding BK blockers into bath solutions. Furthermore, we recorded IK1 currents with ATP present in the pipette solution, which may strongly enhance IK1 activity via a PKC- and/or PKA-dependent mechanism [52], [53]. Unlike Weaver *et al*., we also found weak immunopositive protein bands at the molecular weight that was very close to the predicted molecular weight for the IK1 protein. This band was detected in cell lysates prepared from U251, U87, and primary GBM cells. Such difference in Western blots results may be due to the higher sensitivity of antibody that was used in our study.

The detection of the IK1 subtype of Ca^2+^-activated K^+^ channels in glioma cells may be of substantial importance. These channels have been implicated in tumorogenesis and regulation of cell proliferation in a number of cell types, including pancreatic and endometrial cancer cells, non-malignant endothelial and smooth muscle cells, and bone marrow mesenchymal cells [31], [33], [54], [35]. In *in vivo* experiments, systemic application of the IK1 inhibitor clotrimazole was reported to decrease tumor load and prolong survival in animal models of glioma and melanoma [36], [38]. In our hands, the specific IK1 blockers clotrimazole and TRAM-34 strongly inhibited proliferation of U251 cells. However, as in the case of BK blockers, their effective concentrations strongly exceeded those that were necessary for complete inhibition of the IK1 currents. Moreover, siRNA downregulation of IK1 did not affect cell proliferation. Recently, Sciaccaluga *et al.* discovered a critical role for the IK1 channels in chemotactic motility of glioblastoma cells [51]. In this latter study, downregulation of IK1 expression using shRNA completely inhibited cell migration in response to the chemokine ligand CXCL12 in several glioblastoma cell lines and primary GBM cells, suggesting a possible role of IK1 in glioblastoma invasiveness [51].

Overall, our data provide strong evidence against a requirement for the Ca^2+^-activated K^+^ channels BK and IK1 for proliferation of U251 cells, and likely other glioma cells. Further work is needed to define the mechanisms responsible for the antiproliferative effects of pharmacological inhibitors of BK and IK1, such as paxilline, penitrem A, clotrimazole, and TRAM-34. Our results suggest that strong caution should be taken when interpreting positive results obtained with pharmacological ion channel blockers and that such experiments should be supplemented with molecular biology studies targeting expression of proteins of interest.

## Supporting Information

Figure S1 The BK blocker charybdotoxin (100 nM) potently suppresses whole-cell K+ currents in U251 glioma cells. For methodological details see Methods and legend to Fig. 2 in the main text.
(1.34 MB EPS)

Figure S2 U87 glioma cells express typical IK1 currents that were potently suppressed by the IK1 blocker clotrimazole (2 µM). For methodological details see Methods and legend to Fig. 3 in the main text.
(1.19 MB EPS)

Figure S3 Western blot analysis of the IK1 protein expression in glioma cell lines and primary glioblastoma cells. Protein lysates (20 µg/lane) of primary glioblastoma (GBM), U87, and U251 cells were separated by sodium dodecyl sulfate polyacrylamide gel electrophoresis, transferred to the PVDF membrane and probed with polyclonal anti-IK1 antibody as described in Materials and Methods. Immunopositive signal was detected and digitized using chemiluminescence ECLplus reagent kit (GE Healthcare/Amersham Biosciences) and luminescent image analyzer LAS-4000 (FujiFilm Medical Systems, Stamford, CT). Bars and numbers on the left indicate positions of molecular weight standards (MW). Weak immunopositive band indicated by asterisk was detected at the molecular weight of ∼46–47 kDa that is close to the predicted molecular weight of the IK1 protein (45 kDa).
(0.35 MB EPS)

Figure S4 Effects of paxilline and clotrimazole on proliferation of U251 cells in serum-containing and serum-free media measured using two alternative cell proliferation assays. (A) Effects of paxilline (10 µM) and clotrimazole (10 µM), or their combination on proliferation of U251 in media containing 10% FBS. Cells were treated with pharmacological inhibitors for 48 hrs and rates of proliferation were quantitatively assessed using Coulter Counter (white bars) and MTT (grey bars) assay. Plating cell density is indicated by dashed line. (B) Effects of paxilline (10 µM) and clotrimazole (10 µM) or their combination on proliferation of U251 cells in serum-free media supplemented with serum substitute B27. Cell proliferation was quantified using Coulter Counter (open bars) or MTT assay (grey bars). (C) Effects of paxilline (10 µM) and clotrimazole (10 µM) or their combination on proliferation of U251 cells in serum-free media supplemented with serum substitute B27 and bovine serum albumin. Proliferation rates were measured using MTT proliferation assay. Data are the mean values ±SE of proliferation normalized to controls in the same experiments. **p<0.01 vs. control; **p<0.05 vs control;***p<0.001 vs. control.
(0.41 MB EPS)

Figure S5 Effects of paxilline and clotrimazole on proliferation of U87 cells in serum-containing and serum-free media. (A) Effects of paxilline (10 µM) and clotrimazole (10 µM) or their combination on proliferation of U87 in media containing 10% FBS. Cells were treated with pharmacological inhibitors for 48 hrs and rates of proliferation were quantitatively assessed using MTT assay. Data are the mean values of proliferation ±SE normalized to controls in the same experiments. (B) Effects of paxilline (10 µM) and clotrimazole (10 µM) or their combination on proliferation of U87 cells in serum-free media supplemented with serum substitute B27. Data are the mean values of proliferation ±SE normalized to controls in the same experiments. **p<0.01 vs. control; ***p<0.001 vs. control.
(0.39 MB EPS)

Figure S6 Representative images of U251 cells transfected with the GFP-expressing plasmid using amaxa nucleofection protocol. Images of the same field were acquired using Olympus IX71 Hoffman modulation contrast optics (A) and GFP fluorescence (B) 72 hrs after nucleofection at 10×10 magnification. Transfection efficacy varied between 60–90% in various cell preparations.
(1.24 MB EPS)

Figure S7 Double knockdown of BK and IK1 K+ channels does not affect proliferation of U251 glioma cells. Effect of transfection with negative control (NC), BK-specific siRNA #2 (siBK), IK1-specific siRNA#7 (siIK1) or combination of BK- and IK1-specific siRNAs (siBK+siIK1) on proliferation of U251 measured 72 hrs post transfection using MTT proliferation assay. Data are the mean values ±SE of 3 independent cell transfections.
(0.32 MB EPS)
